# Evaluation of highly adsorptive Guefoams (multifunctional guest-containing foams) as a potential sorbent for determination  of volatile organic compounds (VOCs) by means of thermal desorption

**DOI:** 10.1007/s00604-024-06249-9

**Published:** 2024-02-29

**Authors:** Raquel Sánchez, Ana Beltrán Sanahuja, Lucila Paola Maiorano Lauría, José Luis Todolí, José Miguel Molina Jordá

**Affiliations:** 1 Analytical Chemistry, Nutrition and Food Sciences Department, University of Alicante, P.O. Box 99, 03080 Alicante, Spain; 2 Inorganic Chemistry Department, University of Alicante, P.O. Box 99, 03080 Alicante, Spain

**Keywords:** Guefoam, Thermal adsorption–desorption; Static headspace analysis, Volatile organic compounds (VOCs); Cereal bioethanol analysis

## Abstract

The present work delves into the feasibility of employing a novel structured sorbent referred to as GFAD (Guefoam Adsorption Device) for the determination of volatile organic compounds (VOCs) in liquid samples. The chosen method has been static headspace sorptive extraction-thermal desorption gas chromatography mass spectrometry (HSSE-TD-GC–MS). The GFAD comprises an aluminum cellular material with a distinct replication structure and a solid guest phase consisting of activated carbon particles dispersed within the cavities of the cellular aluminum. The extensive specific surface area, robustness, and exceptional thermal conductivity of this pioneering material offer distinct advantages over commercially available polydimethylsiloxane-based Twister® devices. Therefore, the trapping efficiency for volatile organic compounds is enhanced, and it is possible to perform the analysis of concentrated samples. According to computational simulations, it has been demonstrated that GFAD has a high heat conductivity. As a result, the desorption efficiency is improved, and minimal temperature gradients are generated throughout the GFAD during the heating process. Besides, the energy consumption is significantly lowered, thus aligning with environmentally conscientious and sustainable analytical practices.

The experimental results give a proof of the suitability of the GFAD for determining gaseous compounds in liquid samples through HSSE-TD-GC–MS. For volatile species, the new material provides higher peak areas and lower limits of detection than a commercially available Twister® device. Furthermore, the GFAD is reusable, its adsorbing properties remaining unchanged during, at least, 100 consecutive analyses. In addition, unlike to the Twister®, no intense siloxane peaks are observed in the chromatograms obtained with the GFAD. The feasibility of qualitative and semi-quantitative analysis with the new accessory has been demonstrated with both standards and a cereal bioethanol real sample.

**Graphical abstract:**

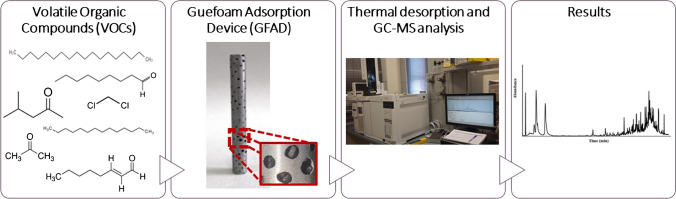

**Supplementary Information:**

The online version contains supplementary material available at 10.1007/s00604-024-06249-9.

## Introduction

Volatile organic compounds (VOCs) are of concern in several areas such as environment, foods, forensic and medical sciences, and even in cultural fields [1–3]. To perform an accurate determination of volatile compounds, sampling methods providing good accuracy and high enough sensitivities must be developed [4].

Solid phase extraction (SPE) is a powerful tool for the analysis of liquid as well as gaseous compounds in complex samples [5]. An accessory named “Twister®” is commercially available in which organic analytes are accumulated on the surface of a polydimethylsyloxane (PDMS) coated bar [6]. Its good thermal stability makes it suitable for the determination of thermally stable compounds through thermal desorption methods [7]. This dispositive can be operated either in a stirred sample or static mode. In both cases, a sample volume is placed in an appropriate container. The Twister® is adapted to the headspace, and volatile compounds diffuse towards the sorbent [6] thus giving rise to the so-called headspace sorptive extraction (HSSE) [8]. Following this step, the bar is accommodated to a thermal desorption tube thus releasing the retained compounds and further leading them to a suitable analysis instrument (e.g., gas chromatograph (GC)).

Among the advantages of the Twister® over other solid-phase extraction techniques such as solid phase microextraction (SPME), its robustness, the superior performances in terms of sensitivity (50 to 250 times higher), and accuracy can be highlighted. In fact, for compounds difficult to extract (*i.e.*, with low partition coefficients), theoretical recoveries are close to 100% and around 40% for the Twister® and SPME, respectively [8]. Note that the total amount of sorbent retained on the bar may range from roughly 20 to 100 µL, whereas only about 0.5 µL is retained on a fiber in SPME [9]. Nonetheless, the latter technique can be more easily automated than the former [6].

HSSE using a Twister® has been successfully applied to the determination of volatile and semi-volatile compounds in different samples [8]. This sampling step on sorbent tubes can be performed according to different methodologies, among them, the gas pumping at a suitable flow rate (i.e., from 0.5 to about 500 mL min^−1^) or passive [10] either axial [11] or radial [12] sampling. In passive sampling, long adsorption times are required without achieving equilibrium. The rapid saturation of the sorbent surface and back-diffusion, especially significant for volatile compounds (i.e., more volatile than benzene [6]), is among the problems encountered with these configurations.

Following a first adsorption step, the retained compounds are thermally desorbed into a carrier gas stream. This procedure is solvent-free and offers a 100% transfer efficiency of the desorbed gases into the column as well as a significant pre-concentration factor. As a result, sensitivities can be improved by around three orders of magnitude over those achieved by conventional liquid–liquid extraction. Automation and avoidance of externally added chemicals are additional reasons for this trend. The released vapors can be separately determined through gas chromatography mass spectrometry (GC–MS), for instance. A further on-line trap can be applied to pre-concentrate the analytes before their injection into the GC column.

Activated charcoal followed by CS_2_ extraction has been used for the determination of non-polar compounds, although they afford low sensitivities, and the adsorption yield differs depending on the compound [13]. This material has characteristics (hydrophobicity, reactivity) that make prohibitive its use in thermal desorption. To expand the field of applicability of this technique, multiple sorbents (typically from 2 to 4) can be used for the analysis of compounds with different properties [6, 7]. Problems related with the reactivity, water retention, or the fact that multicomponent sorbents are only suitable to perform active sampling have been claimed.

In order to select the best sorbent, several properties must be considered: (i) strength, the sorbent should retain the analytes under the sampling step and release them at the desorption temperature. If the sorbent is too weak, big tubes will be required thus leading to wide peaks, tedious conditioning and purge protocols, increased likelihood for analyte oxidation and complex blanks; (ii) inertness; (iii) hydrophobicity; (iv) absence of artifacts; (v) thermal and mechanical stability. Current sorbents include [16] porous polymers (e.g., Tenax®, Chromosorb 106, UniCarb, or Carboxen 1003), strong and hydrophobic graphitized carbon blacks [14], sorbents based on nanoparticles [15], sorbents impregnated with derivatizing agents [6], or molecularly imprinted polymers [16].

The most important problem associated to static mode Twister bar extraction lies in the poor adsorption efficiency for polar compounds although possible strategies such as derivatization have been proposed [9]. Additional problems can be related with the limited number of coating materials commercially available (*i.e.*, mainly PDMS and poly(ethylene glycol) in PDMS). The large volume of coating and the high viscosity of these two phases lead to analyte diffusion during extraction, resulting in a longer extraction and desorption time (typically between 30 and 240 min [6]) in comparison with SPME [10, 17].

The most widely used approaches for HSSE are based on the retention of the analytes on the sorbent surface. Obvious physical constraints avoid the work with sorbents with high specific surface areas. This may cause inefficient retention of gaseous compounds and/or increase the fragility of the sorbent device. Furthermore, the employed materials have low thermal conductivities that may hamper the rapid desorption of the analytes of interest. The aim of the present work was thus to develop and characterize, for the first time, a novel sorbent bar consisting of a metallic foam as a support for the sorbent that was dispersed as solid particles inside the foam cavities. An increased heat conduction through the metallic net was expected thus giving rise to short desorption times and minimizing radial temperature gradients. Furthermore, as the adsorption phase, *i.e.*, active carbon, was dispersed into the foam, the entire active surface was exposed to the sample thus giving rise to an increase in both the adsorption and desorption efficiencies.

## Experimental procedures

### Materials

High-purity aluminum (99.999 wt%) was purchased from Goodfellow Cambridge Ltd. (UK) and used as a precursor of the matrix foam. Nuchar RGC-30-activated carbon particles from Westvaco Chemical Division (Covington, USA) with a diameter of 0.5 to 1.0 mm were selected as sorbent/guest phase. Sodium chloride (99.5% purity) was supplied by Panreac Química S.L.U. (Barcelona, Spain) and used for coating the guest phase. The resulting structure was called Guefoam Adsorption Device (GFAD). Additionally, a commercially 1-cm-length 1-mm thickness Twister® for HSSE with PDMS phase was purchased from Gerstel GmbH & Co. KG (Mülheim an der Ruhr, Germany).

### Reagents and samples

Nonanal, trans-2-octenal (analytical standards), and dichloromethane were supplied by Merck KGaA (Darmstadt, Germany). Ethanol and acetone (GC grade) were purchased from Scharlab S.L. (Sentmenat, Spain). Dichloromethane, methyl isobutyl ketone (MIBK), o-xylene, and toluene were purchased from Panreac S.A. (Barcelona, Spain), and propan-2-ol solvent (GC grade) was purchased from Labkem (Barcelona Spain). Decane, dodecane, and tetradecane (analytical standards) were supplied by Aldrich (Steinheim, Germany). A real bioethanol sample obtained from a winemaking residue was analyzed to validate the developed GFAD.

### Fabrication of the GFAD material

**Fig. 1:**
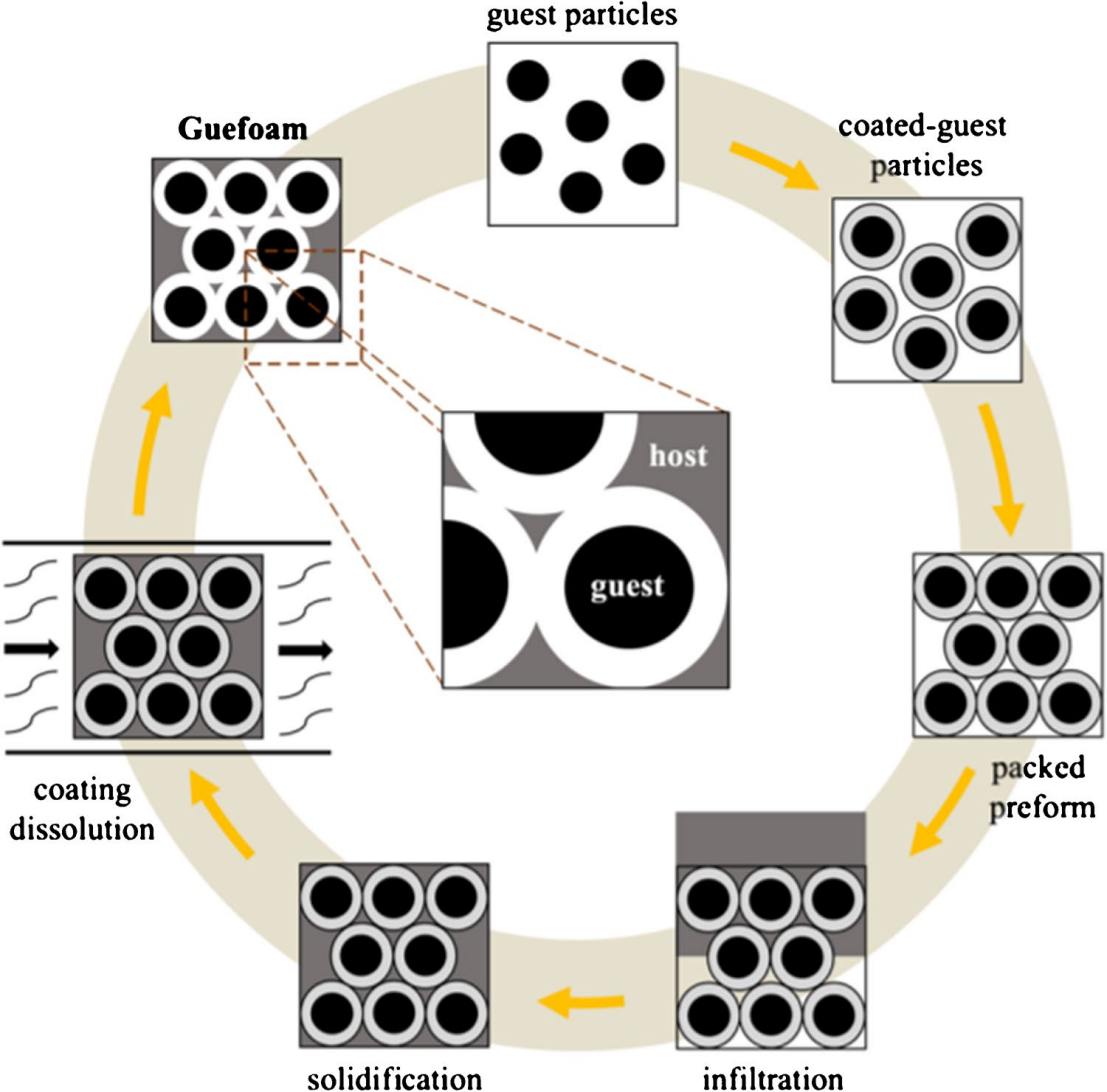
Schematic diagram of the fabrication procedure of the GFAD material

The GFAD material was prepared by the gas pressure–assisted liquid metal infiltration technique according to the replication method [18, 19]. Activated carbon (Ac) particles were coated by spray deposition with a 20 wt% aqueous solution of sodium chloride (NaCl) as described in [20–22]. As a result, quasi-spherical NaCl-coated activated carbon particles with an average diameter of 1.0–1.5 mm, hereafter referred to as NaCl-coated Ac particles, were obtained. These were carefully packed into a graphite crucible with an inner diameter of 3 mm and a length of 50 mm using gentle vibration. The top of the packed porous preform was sealed with a 2-mm-thick graphite disk with 0.2 mm holes to prevent particle movement during infiltration. A solid piece of aluminum was placed over the graphite disk, and then, the crucible was inserted into a chamber designed for pressure infiltration. The vacuum in the chamber was lowered to 0.2 mbar, and the crucible was subjected to a heating rate of 4.5 °C min^−1^. After 10 min at a constant temperature of 750 °C, the vacuum was closed, and infiltration of the liquid aluminum into the packed preform was achieved by applying 0.8-bar Ar. The solidification of the metal was accomplished by rapid directional cooling of the chamber in a water bath at room temperature. The infiltrated material was then removed from the crucible and carefully polished. The final step of the Guefoam fabrication process was the removal of the NaCl coating on the guest phase particles by dissolution in water [23]. As a result, a material was obtained consisting of an interconnected porous Al matrix (host phase) containing Ac (guest phase) in its porous cavities, without any bonding with the matrix other than mere physical contact. The Guefoam fabrication process is schematically shown in Fig. 1.

### Specific surface area and structural characterization of the sorbent phases

Nitrogen adsorption isotherms were performed at − 196 °C in a 3FLEX system (Micromeritics) equipped with 1.5 × 10^−4^ bar sensors that allowed high-resolution isotherms. The specific surface area of the sorbents was determined via the Brunauer–Emmett–Teller (BET) method using desorption data from isotherm branches.

The microstructure of the NaCl-coated Ac particles was characterized by scanning electron microscopy (SEM-Hitachi S3000N) to verify the continuity of the NaCl coating layer and the absence of cracks. In addition, materials were observed under an optical microscope (Olympus PME-3) after dissolution of the NaCl coating. Geometric descriptors such as Feret minimum, Feret maximum, and aspect ratio of the Ac and NaCl-coated Ac particles were determined by image analyses. The specific surface area of the Ac particles was obtained from the nitrogen adsorption isotherm at − 196 °C (Autosorb 6, Quantachrome Instruments). The pore volume fraction and the parameters known as guest loading and guest occupation (see Eqs. 1–2) were determined by densitometry and analytical calculations, which are described in the following sections.

### Extraction of volatile organic compounds by using GFAD

Prior to their use, GFADs were conditioned at 300 °C for 120 min under nitrogen atmosphere. To evaluate the performance of the GFAD, a 0.01 mg kg^−1^ nonanal and trans-2-octenal solution (of recent interest in the food industry [24, 25]) was prepared in hexane. Afterwards, the aldehyde mixture (3 mL) was introduced inside a closed magnetically stirred 20 mL vial with the GFAD in the headspace (0.7 cm of the GFAD bar remained outside of the vial). The vial was heated at 60 °C for 30 min after selecting a 10-min equilibration time. This time was similar to that set in other studies in which SPME fibers were used for the determination of volatile compounds in gas samples [26]. Note that in the case of food applications, it has been observed that the adsorption time can be as long as 5 h, depending on the sample to be analyzed [27]. Once this step was completed, the cartridge was adapted to the desorption unit, and the temperature was set at 300 °C for 1 min.

In addition, two different solutions containing a series of organic solvents present at the same proportion were evaluated: solution 1 contained ethanol, propan-2-ol, toluene, and xylene, whereas solution 2 consisted of a mixture of acetone, dichloromethane, isobutil-methyl-ketone (MIBK), decane, dodecane, and tetradecane. To perform the study, 100 nL of each organic mixture was placed inside the vial. The container was heated at 60 °C to promote the complete solution evaporation, and the vessel was left for 10 min to allow the equilibration of its atmosphere. Then, the GFAD was introduced into the vessel and carefully hanged for 15 min to promote the efficient retention of the volatile solvents. Once this step was completed, the desorption was followed at 250 °C and the chromatogram finally registered. The same experimental conditions were employed in the analysis of the bioethanol real sample by using the developed GFAD.

Four GFADs of different lengths (*i.e.*, 0.5, 1, 3.2, and 4.2 cm) were prepared and adapted hanging at the uppermost part of the 20-mL inner volume glass container. Afterwards, the glass vial containing 100 nL of the mixture was heated at 60 °C for 15 min for all the experiments. In this way, the sorbent was in static contact with the vapors of the sample during the extraction step. A commercially available 1-cm-length Twister® device was used for comparative purposes.

### Thermal desorption

The sample introduction system consisted of a thermal desorption unit (TDS-2) equipped with a programmed temperature vaporization (PTV) cooled injector system (CIS-4 +) by Gerstel (Mülheim an der Ruhr, Germany). The thermal desorption unit was operated in splitless mode. The desorption temperature was programmed from 35 to 350 °C; the ramp rate was 60 °C min^−1^, and the helium flow was 100 mL min^−1^. The PTV system was programmed from − 50 to 250 °C and held for 3 min at 10 °C s^−1^ prior to GC–MS analysis. The desorbed analytes were then cryofocused in the PTV system using liquid nitrogen at − 50 °C.

### GC–MS analysis

The TDS system was attached to an Agilent 6890N Gas Chromatography System coupled with an Agilent 5973N Mass Spectrometry Detector (Agilent Technologies, Palo Alto, CA, USA). The separation was achieved on a DB-624 column (J&W Scientific, Folsom, CA, USA), 30 m × 250 µm × 1.4 µm. Helium was used as carrier gas, and its flow rate was maintained at 1.4 mL min^−1^. The oven program for GC was started at 35 °C, held for 10 min, then ramped up to 100 °C at 5 °C min^−1^ and held for 1 min, then increased to 250 °C at a rate of 10 °C min^−1^ and, finally, held for 10 min. The mass spectrometer was operated in electron ionization mode at a voltage of 70 eV in a range from 30 to 450 amu. Peaks obtained from scanned mass spectra were identified by matching with the Wiley 725 Edition Library (Wiley Registry of Mass Spectral Data, 2000).

### Computational simulations

**Fig. 2:**
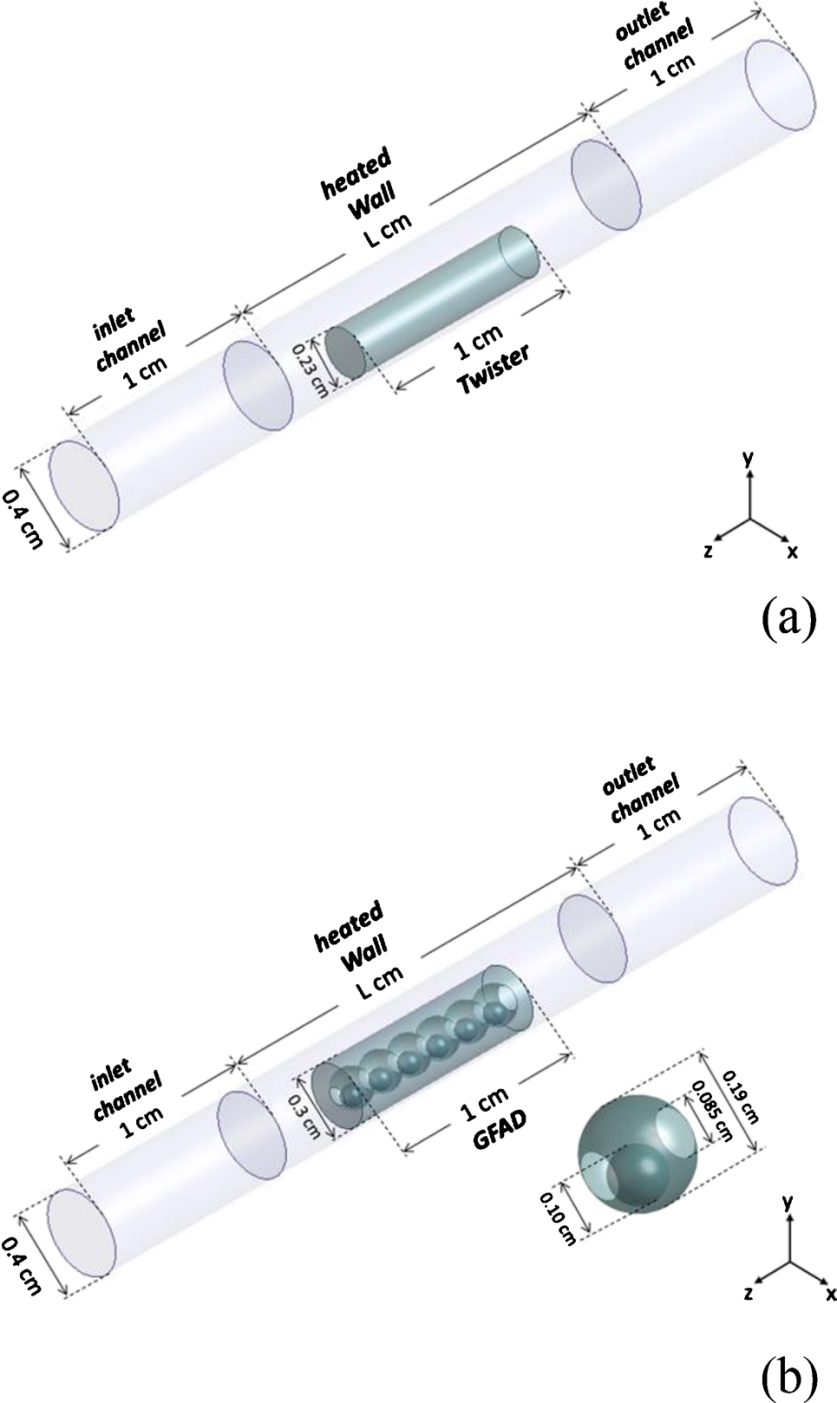
Schematic representation of the 3D domains and their dimensions employed for the simulations of a Twister® (**A**) and a GFAD (**B**)

Thermal and fluid-dynamic computational simulations were conducted employing the commercial software Ansys-Fluent. The three-dimensional domains under investigation, along with their corresponding dimensions, are schematically presented in Fig. 2. The configuration faithfully replicates that of the experimental setup, wherein the fluid is channeled through a conduct containing the material under examination. The heated wall has a variable length *L* (*L* = 18 cm and *L* = 2 cm for simulations focusing on studying experimental conditions and energy-efficient conditions, respectively). The structural complexity of both materials was intentionally streamlined. The thermophysical attributes linked to both solid and fluid domains (Table 1) are assumed to remain constant throughout. The fluid flow is assumed to be incompressible and maintains a laminar behavior. The boundary conditions applied to the computational domains are as follows: (i) a uniform fluid velocity at the inlet (0.133 ms^−1^, corresponding to the experimental flow rate of 100 mL min^−1^); (ii) maintenance of zero pressure-gauge at the outlet; (iii) imposition of a variable temperature boundary along the heated wall (ranging from 25 to 350 °C at a ramp rate of 60 °C min^−1^). In contrast, a prescribed constant heat flux (2500 Wm^−2^) is applied when assessing energy efficiency, and (iv) adiabatic characteristics are ascribed to walls within the inlet and outlet channels. The numerical analysis was conducted using a transient pressure-based method. The residual values for the energy and momentum equations were defined to be on the order of 10^−9^.

**Table 1:** Thermophysical properties of the solid and fluid domains employed for the computational simulations. ρ, Cp, and *k* denote the density, heat capacity, and thermal conductivity of the materials, respectively

	Solid domains			Fluid domain
Material	Twister®	GFAD matrix (aluminum)	GFAD guest phases (activated carbon)	Helium
ρ (k gm^−3^)	2230	2700	700	0.16
*Cp* (J kg^−1^ K^−1^)	795	897	2310	5193
*k* (W m^−1^ K^−1^)	1.20	237	0.17	0.15

## Results and discussion

### Selection of experimental conditions based on computational simulations

The present study undertakes a comparative analysis of the sorption–desorption dynamics between a newly developed GFAD and a commercial Twister®. The latter device contains a non-permeable material whose functionality relies only on its external surface so that the carrier gas can surround it but not flow through it. Conversely, the high permeability of the GFAD enables gas penetration, thereby fostering interaction with guest phases, specifically in the case of components endowed with sorbent capabilities.

Critical to our experimentation are the selected operational conditions, encompassing carrier fluid velocity, and heating ramp. These parameters must be thoughtfully chosen to ensure that during the desorption procedure, (i) material temperatures rise uniformly and (ii) no significant temperature gradients emerge between the hotter and colder regions of each material. While operating desorption conditions, modest in terms of gas flow and temperature rise, seemingly fulfill the prerequisites, their appropriateness was subjected to validation via computational fluid dynamic (CFD) simulations.

Fig. S1 shows the temporal temperature of two specific points on the upper surface of the adsorbent phases in relation to the pre-set temperature profiles of the container tube walls. These points are positioned at length coordinates *z* = 0.085 cm (front region) and *z* = 0.925 cm (rear region). Both stipulated requirements are impeccably satisfied: neither material exhibits discernible delays in temperature rise or temperature gradients remain absent. Despite identical thermal conditions, the divergent microstructures of the two materials give rise to substantial variations in their sorption capacities. Fig. S2 illustrates the contrasting fluid velocity profiles of the two configurations within the proximity of the sorbent material once the prescribed maximum temperature (350 °C) has been attained.

### Characterization of the sorbent phases (twister and RGC-30 activated carbon particles)

**Fig. 3:**
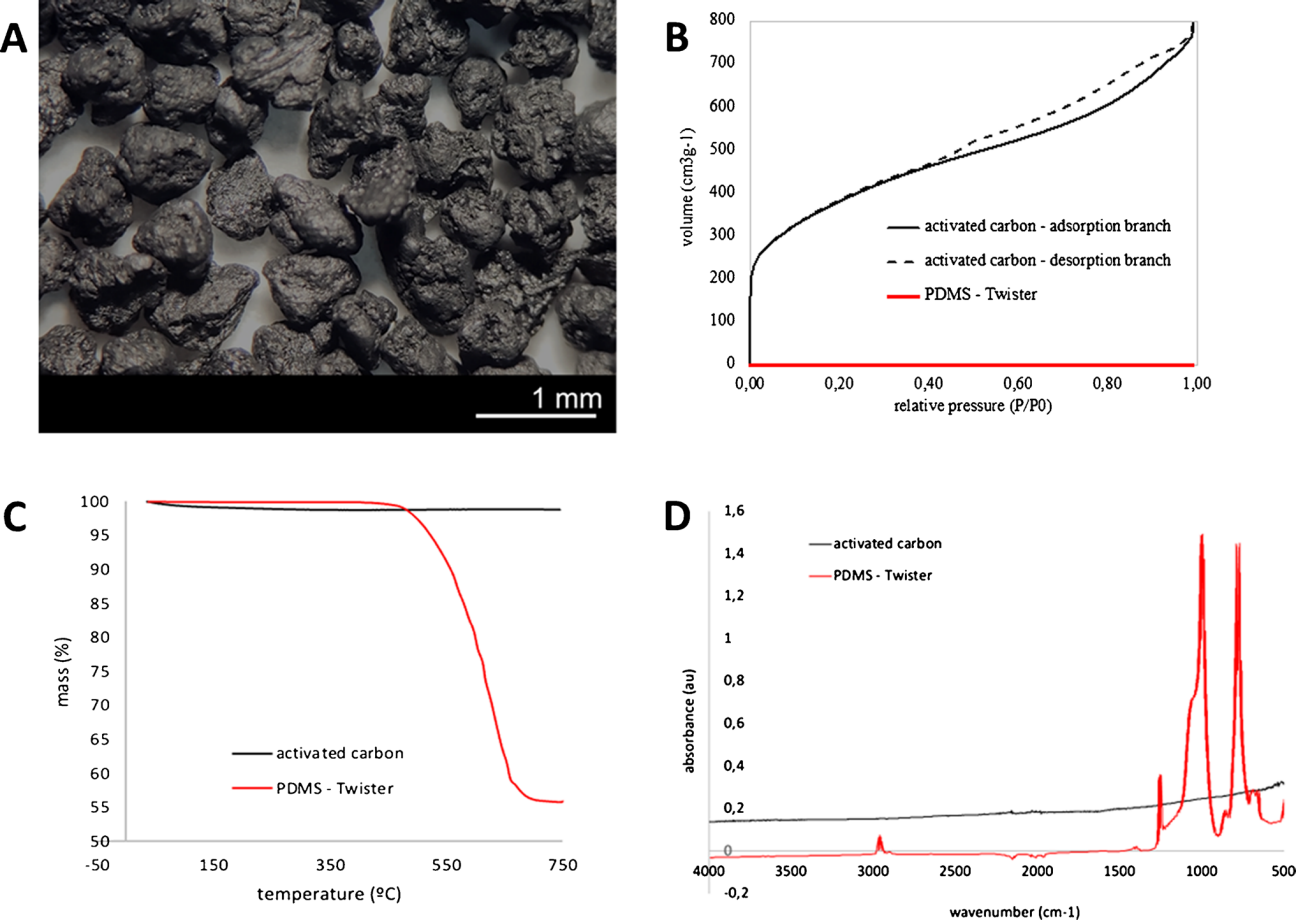
Activated carbon particles (**A**); nitrogen adsorption–desorption isotherms at –196 °C for the sorbents used in this study: activated carbon RGC-30 and PDMS Twister (**B**); thermogravimetric curves for both sorbents (**C**); and FTIR spectra (**D**)

Figure 3A depicts a photograph of the activated carbon particles in their original state, showing their irregular surface texture and rounded geometrical structure. The nitrogen adsorption–desorption isotherms at − 196 °C for the two distinct materials being investigated are shown in Fig. 3B. At first glance, a marked contrast between these materials becomes apparent. The Twister material exhibits no measurable nitrogen adsorption at any pressure, thus classifying it as a non-porous polymeric substance. This observation aligns with the underlying principle that governs gas retention in this material, which is absorption rather than adsorption. As a result, significant physisorption processes can be ruled out. In sharp contradistinction, the RGC-30 activated carbon particles manifest an adsorption profile typical of a porous material. This profile is characterized by the combination of micro- and mesoporosity, with a discernible hysteresis loop initiating at a relative pressure of approximately 0.4. It is important to note that in the lexicon of adsorption, the designations “micropores” and “mesopores” denote pores with average sizes of less than 2 nm and within the range of 2–50 nm, respectively.

According to the BET method, the RGC-30 activated carbon particles exhibit a remarkable specific surface area, quantified at approximately 1295 m^2^ g^−1^. Although the specific surface area of the Twister® material is nominally zero and it is not a porous material that allows the physical phenomena of adsorption–desorption, the phenomenon of volatile outflow with temperature is referred to as thermal desorption to improve readability in the present work.

The evaluation of thermal stability of activated carbon particles and PDMS was pursued through rigorous thermogravimetric analysis, as illustrated in Fig. 3C. The activated carbon material remains stable over the entire temperature range tested, bearing a modest maximal mass loss of 1.5%. Since this mass loss starts at low temperatures, it is probably due to the release of adsorbed species rather than the removal of volatiles from reactions of the carbon material with functional groups on its surface. Conversely, an alternate vista unfolds for the PDMS material, revealing an incipient temperature of thermal decomposition of approximately 450 °C. A conspicuous 48% total mass depletion at 750 °C is tallied for PDMS. Elucidating this divergence, Fig. 3D presents the Fourier-transform infrared spectra (FTIR) of both materials. In consonance with the results of the thermograms, the carbonaceous material exhibits a discernible dearth of significant surface functional groups, while PDMS showcases two distinct bands at approximately 780 and 1000 cm^−1^, corresponding with Si-CH_3_ and Si–O-Si bonds, respectively. These spectral features emphatically underscore the pronounced silicon composition characterizing the PDMS material.

### Microstructure and macrostructure of GFAD

**Fig. 4:**
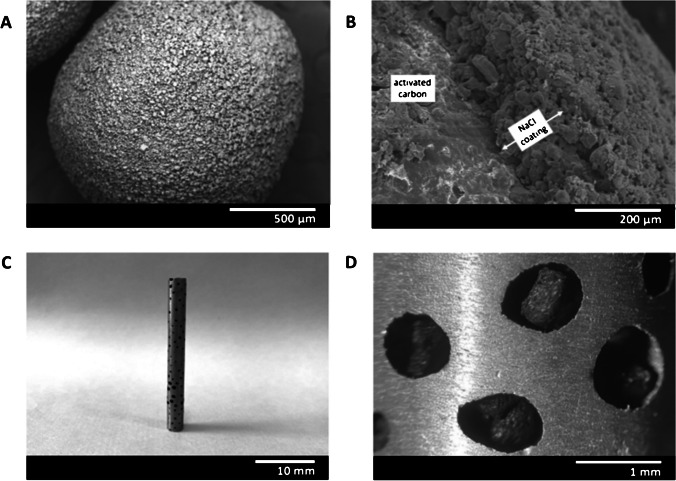
**A**, **B** SEM image of a quasi-spherical Ac particle coated with NaCl, **C** a photograph of the resulting Guefoam material, and **D** a magnification of **C** showing the guest Ac particles contained in the cavities of the porous aluminum matrix

Figure 4A shows a scanning electron micrograph of the microstructure of a NaCl-coated Ac particle. The image confirms a continuous, uncracked coating produced by NaCl spray deposition. The thickness of the coating is observed in Fig. 4B on a particle that was intentionally fractured. Figure 4C depicts an image of the resulting Guefoam material after NaCl dissolution. Further magnification in Fig. 4D shows that the porous aluminum structure hosts activated carbon particles in its cavities, with a space gauge between them and the matrix.

The volume percentage of guest phase present in the porous cavities can be defined as the guest occupation, while the guest loading describes the percentage of pores with guest phase. Both parameters follow the equations [25]:

1 $$\text{GO}=100\times\frac{V\;\mathrm{guest}\;\mathrm{phase}}{V\;\mathrm{hosting}\;\mathrm{pore}}$$

2 $$\text{GL}=100\times\frac{n\;\mathrm{hosting}\;\mathrm{guest}\;\mathrm{phase}}{n\;\mathrm{total}}$$

where GO is the guest occupation, GL is the guest loading, *V* refers to volume, and *n* refers to the number of pores.

The particle size distributions of the guest phase (Ac particles) and NaCl-coated guest phase (NaCl-coated Ac particles) are shown in Fig. S3. The distributions were fitted to Gaussian functions from which the mean diameters were determined. To determine the guest occupation, mean diameters of 0.948 and 1.256 mm were derived from the minimum and maximum Feret values for Ac and NaCl-coated Ac particles, respectively. Assuming spherical geometry for both particles, Eq. (1) leads to a value of GO = 43%. Likewise, derived from subtracting the mean diameters of the NaCl-coated Ac and Ac particles, the thickness of the NaCl layer is about 154 µm. This value can be verified from the detailed micrograph in Fig. 4B. The guest loading was also determined by dissolving a total of 200 NaCl-coated Ac particles. Of these, only 4 contained no guest phase, 6 contained 2 Ac particles, and the rest contained a single Ac particle. According to Eq. (2), it was found that GL = 95%.

### Feasibility of the GFAD as adsorption–desorption media for volatile compounds

#### Evaluation of the retention-desorption capability of GFAD for nonanal and trans-2-octenal

Firstly, the adsorption/desorption process of nonanal and trans-2-octenal (two compounds of interest for the food industry) was characterized by means of the comparison of the GC–MS chromatograms obtained by using the developed GFAD bars. Two well-defined chromatographic peaks were obtained at 13.0 and 13.4 min that were attributed to trans-2-octenal and nonanal, respectively.

To verify that the totality of these compounds left the adsorption phase, a second desorption cycle was applied at 300 °C. It was confirmed that the respective peak areas were less than 1% and 5% of those obtained after the first desorption for trans-2-octenal and nonanal, respectively.

As regards the precision of the procedure, three different GFADs were produced and used. For nonanal, the obtained mean peak height was 2.3 × 10^8^ counts whereas the standard deviation (*n* = 3) was 0.3 × 10^8^ counts. This corresponded to an RSD = 14% which revealed that both the production process and the analysis were acceptably repeatable.

#### Validation of the GFAD performance with solvent mixtures

Further experiments were conducted with two different solutions containing 100 nL of several organic compounds of interest for areas such as air, water, and soil VOC determination [28]. The solutions contained alcohols, ketones, and aromatic as well as non-aromatic hydrocarbons [29]. Table S1 summarizes the compounds tested in the two evaluated solutions together with their chromatographic retention times and boiling points. The GFAD was able to retain light (*e.g.*, ethanol) as well as heavier (*i.e.*, xylene) compounds that were subsequently desorbed. This fact was verified with a more complex mixture containing ketones and hydrocarbons (Table S1) thus suggesting the suitability of the novel GFAD for VOC determination through HSSE-TD-GC–MS.

### Evaluation of the performance of the GFAD under different operating conditions

#### Effect of the desorption temperature

**Fig. 5:**
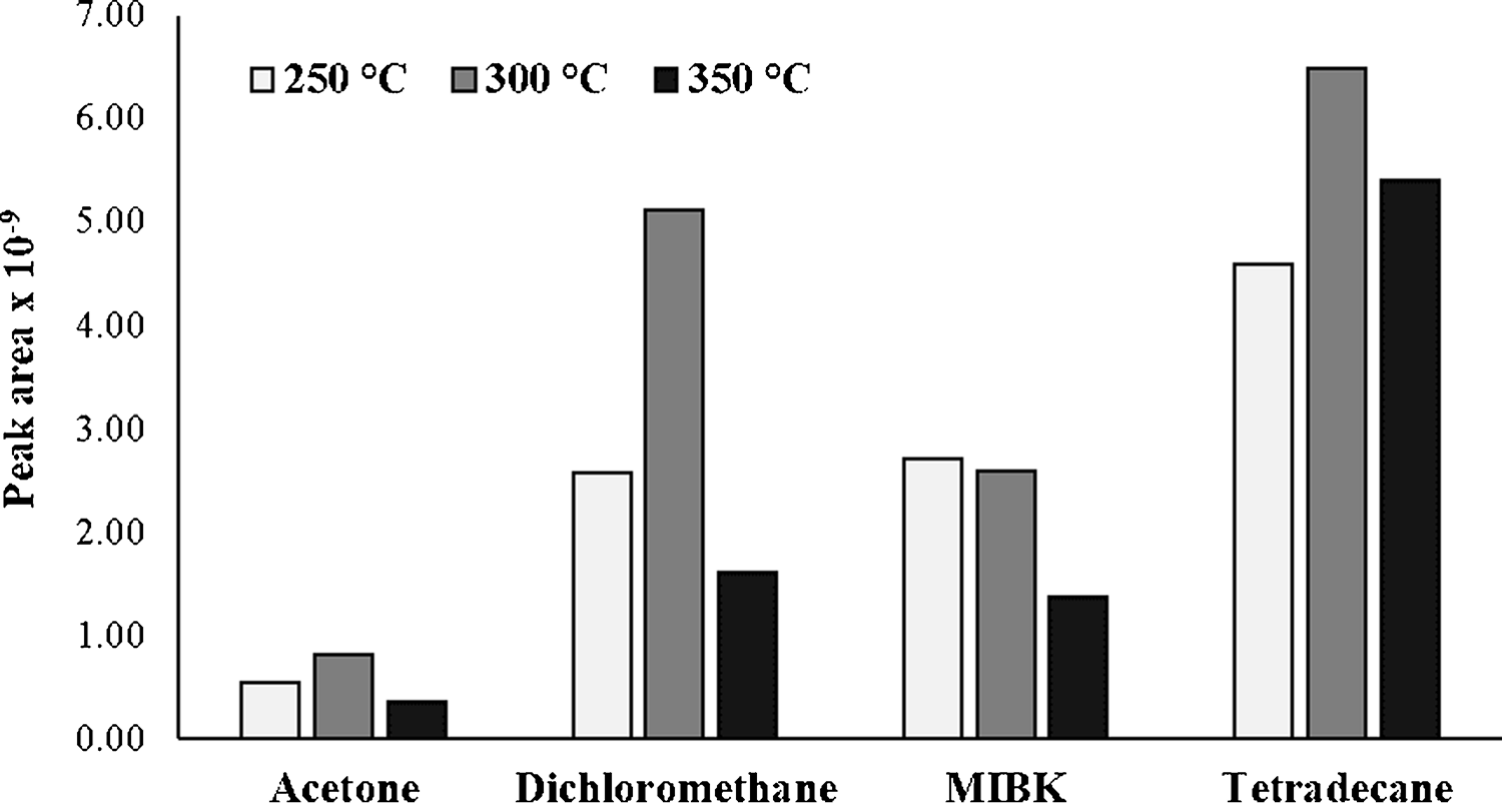
Effect of applying different desorption temperatures (250, 300, and 350 °C) on the peak area obtained with the GFAD for acetone, dichloromethane, MIBK, and tetradecane

A study was performed to verify whether once adsorbed, the volatiles were effectively desorbed. Three different desorption temperatures (250, 300, and 350 °C) were tested. These temperature values were selected for comparison purposes because, according to previous works, the use of a Twister® at higher temperatures gave rise to the release of siloxanes (hexa-methyl-cyclotrisiloxane, octa-methyl-cyclotetrasiloxane, deca-methylcyclopentasiloxane, and tetradeca-methyl-cycloheptasiloxane) as thermal degradation products [30]. Figure 5 reveals that for a given compound, an increase in this variable from 250 to 300 °C led to an increase in the peak area, thus suggesting that the desorption of the organic compounds was favored for the latter temperature.

The aforementioned trend was not observed in the case of MIBK for which an increase in desorption temperature from 250 to 300 °C induced a marginal decrease in peak area (Fig. 5). To understand this observation, it could be indicated that as it has been previously anticipated, the adsorption and stability of MIBK on activated carbon depend, among other variables, on its concentration, the temperature of the medium, and sorbent characteristics such as carbon type and water content [31, 32]. Therefore, a possible hypothesis was linked to the MIBK decomposition on the sorbent surface that could be catalyzed by the reactive carbon sites. In fact, some authors claim that low temperatures (*c.a.*, 4 °C) are better suited than room temperature to stock MIBK on activated carbon [32]. This point, together with the efficient MIBK adsorption on the GFAD surface, should be carefully considered in order to understand the performance of the sorbent and to further optimize it.

Finally, as Fig. 5 reveals, a further increase in temperature from 300 to 350 °C yielded a drop in peak area that was less severe for the least volatile of the studied compounds (*i.e.*, tetradecane). This could be attributed to the poor trapping of non-volatile compounds and/or their stability during the desorption step. In any case, analyte-dependent decomposition at 350 °C on the activated carbon surface could not be ruled out. From these results, it was concluded that 300 °C was the optimum desorption temperature. This value agrees with previous works in which tetradecane was analyzed by using a Twister® bar coated with 24 µL of PDMS (length, 1 cm) [33]].

#### Effect of the GFAD length

**Fig. 6:**
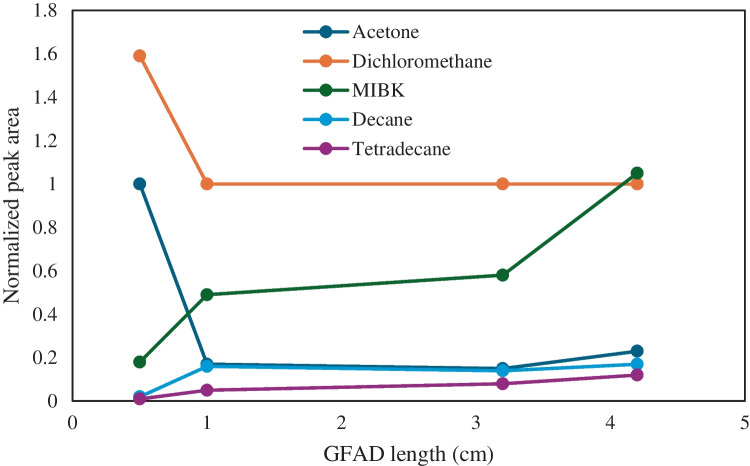
Impact of the GFAD length on the normalized peak area for acetone, dichloromethane, MIBK, decane, and tetradecane. Desorption temperature, 300 °C

The length of the GFAD played a critical role because both, the adsorption and desorption yields, were precluded by this variable. The obtained results are shown in Fig. 6 for the studied compounds: acetone, dichloromethane, MIBK, decane, and tetradecane. Two different trends emerged depending on the organic compound under study. For the most volatile ones (*i.e.*, acetone and dichloromethane), a 0.5-cm tube length provided the best results in terms of normalized peak area. Meanwhile, for the remaining compounds whose boiling points ranged from 116 °C (MIBK) to 254 °C (tetradecane), the longer the tube, the higher the peak area. This trend can be understood by considering that highly volatile compounds were more easily retained on the GFAD whereas heavier compounds required a longer GFAD to be efficiently trapped. This could agree with the fact that as a polar substrate was used to trap the compounds, those with a higher polarity were more efficiently retained than the least polar ones.

The degree of desorption of the different compounds was evaluated by applying an additional desorption program. Interestingly, the most volatile species (acetone and dichloromethane) left the bar after the second desorption process. Nonetheless, the less volatile compounds remained in the GFAD after the second desorption. Thus, in the case of decane, once completed the second desorption process, a chromatographic peak was found to have an area that was approximately 50% of the area measured after the initial desorption. This fact confirmed that the least volatile compounds were more difficult to elute than compounds having low boiling points.

#### Comparison of the GFAD with a conventional Twister®

The activated carbon used in the GFADs developed in the present study had a specific surface area of 1295 m^2^ g^−1^. Therefore, for the 1-cm-length Guefoam bars, the total surface was 3 m^2^, whereas for the 3.2-cm-long bar, the surface was 9.7 m^2^. These figures were in marked contrast with the Twister® bar surface (0.013 m^2^). In other words, the GFAD respective surface areas were 230 and 750 times higher than for the conventional Twister®. This highlighted the potential benefit of the developed bars as adsorbing media for the determination of volatile compounds in gaseous environments.

To evaluate the potential use of our approach, the results provided by the GFAD were compared against those afforded by a conventional Twister® device. Several observations were made: (i) it was observed that the peak areas of the compounds eluting at low retention times (i.e., acetone, dichloromethane, and MIBK) were higher when using the GFAD than for the Twister®; (ii) when the Twister® was used, a wide band was found at retention times in between 25 and 36 min. This band was much more intense than that registered for the GFAD, thus suggesting that the former design was more prone to contamination than the latter; and (iii) it was verified that defined intense peaks appearing after the second desorption cycle corresponded to siloxanes originating from either the column or the Twister®.

**Fig. 7:**
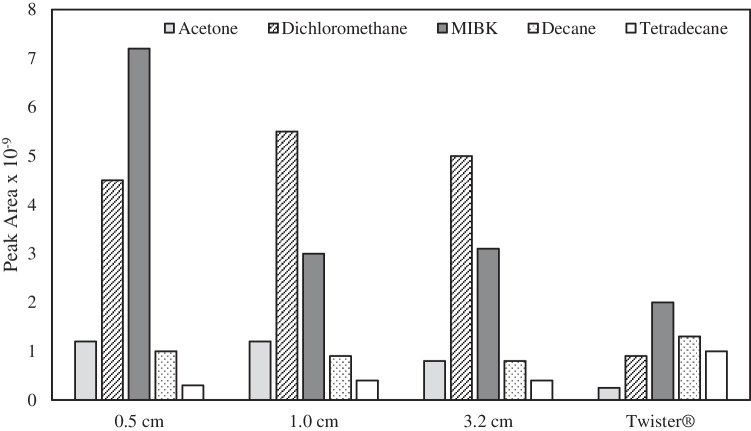
Comparison of the values for the peak areas of acetone, dichloromethane, MIBK, decane, and tetradecane from the chromatograms obtained by using the conventional Twister and the developed GFADs of different lengths (0.5, 1.0, and 3.2 cm)

As regards peak areas, Fig. 7 establishes a comparison between the data obtained for the Twister system and those for three GFADs having different lengths (0.5, 1.0, and 3.2 cm). It may be observed that for the three most volatile compounds, the new device provided peak areas 3–4 (acetone), 6–8 (dichloromethane), and 1.5–4 (MIBK) times higher than the Twister®. In the case of decane, similar peak areas were found with both devices, whereas for tetradecane, the Twister® gave rise to peak areas two times higher than the GFAD. Note that the boiling points for these two compounds were 174 and 254 °C, respectively. Therefore, by considering that only 50% of the mass of this compound was released in the first desorption step when using the GFAD, it was concluded that for non-volatile compounds, the Guefoam bar could potentially provide results similar to those encountered with the Twister® bar. It has been observed that unlike for other more volatile compounds, the mechanism for decane and tetradecane desorption from activated carbon is based on a partial decomposition of these alkanes [34]. This would also explain possible losses of these compounds during the release step at 350 °C. It thus emerged that for these compounds, either the new accessory design or the operating conditions (*i.e.*, desorption time) had to be optimized.

An interesting observation was also made, because, after the desorption was completed, in the case of the Twister, several peaks were found at 17 and 25 min retention times whose height was 44 and 66 times higher than for the GFAD, respectively. Furthermore, for the conventional system, intense peaks appeared at 29, 32, 34, and 36.5 min that were not observed for the Guefoam bar. Their mass spectra revealed that all these peaks corresponded to silicon-based compounds. Thus, it was concluded that, unlike the GFAD, the PDMS Twister partially degraded during the desorption step.

Limits of detection (LOD) were calculated according to the following equation:

3 $${\text{LOD}}= \frac{3 {s}_{{\text{b}}}}{S}$$

where *s*_b_ was the standard deviation of 10 consecutive blank measurements, and *S* was the sensitivity defined as peak area divided by concentration.

Table 2 summarizes the LODs found for the different compounds tested, the GFAD and the Twister®. As expected from the sensitivity results, it was found that for the most volatile species, the new sorbent exhibited limits of detection from around 2 to 8 times lower than the conventional device, thus highlighting the GFAD capability for VOC trace analysis. As observed in terms of sensitivity (Fig. 7), in the case of less volatile compounds, the GFAD afforded LODs similar to or higher than the reference accessory. A further optimization of the operating conditions and/or rod design could improve the results, also for these species.

**Table 2:** Comparison of limits of detection obtained with the GFAD against those provided by the Twister® device

Compound	LOD GFAD (μg/L)	LOD Twister® (μg/L)
Nonanal	2.4	2.5
Trans-2-octenal	0.3	0.5
Ethanol	2.1	8.4
Propan-2-ol	1.0	3.0
Toluene	33	98
o-Xylene	46	92
Acetone	121	484
Dichloromethane	252	202
MIBK	24	96
Decane	184	202
Dodecane	62	65
Tetradecane	274	164

### Performance of the GFAD in the analysis of a real bioethanol sample

As a proof-of-concept, a real bioethanol sample was analyzed by means of the GFAD. Once produced, this biofuel may contain many organic pollutants [35]. These chemicals negatively affect the bioethanol quality since they degrade the combustion yield, the engine performance, and the catalyst efficiency. In addition, some of these species may induce the degradation of the ethanol itself, and harmful gaseous emissions may be produced.

**Fig. 8:**
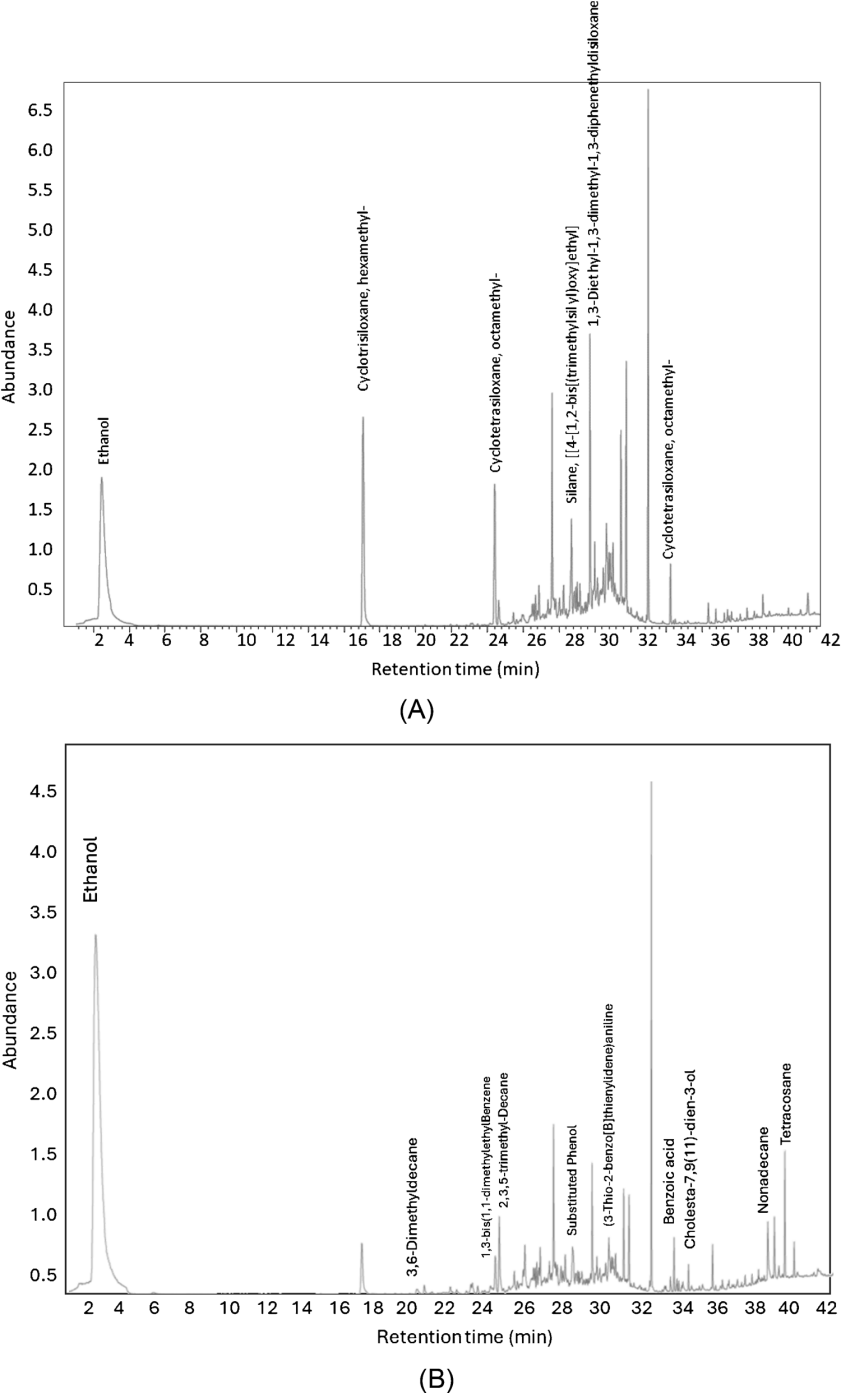
GC chromatograms obtained for the real bioethanol sample under optimum conditions. **A** Twister® and **B** GFAD

The obtained GC–MS chromatograms are shown in Fig. 8. It was observed that as mentioned above, in the case of the Twister® (Fig. 8A), a high number of peaks were found at retention times in between 24 and 34 min that corresponded to silanes and siloxanes. These peaks were superimposed over an intense wide band. Obviously, this fact hampered the correct peak identification of bioethanol trace compounds. In the case of the GFAD, instead, the chromatogram was much cleaner, and hence, minor species were more easily identified. Figure 8B shows some of the compounds detected in the bioethanol sample.

A total of 33 different compounds were clearly identified; among them were 5 alcohols, 16 hydrocarbons, 7 aromatic compounds, 3 ketones, 1 aldehyde, and 1 acid. The list of the most abundant encountered compounds is summarized in Table S2.

Bioethanol semiquantitative analysis was performed by using standards containing a mixture of alkanes (octane, decane, dodecane, and tetradecane), alcohols (ethanol and propan-2-ol) ketones (acetone and MIBK), and aromatic compounds (xylene and toluene). The evaluated concentration was in between 0.2 and 160 mg organic compound per liter. A good linear relationship was observed between peak area and analyte concentration for all the evaluated compounds (*R*^2^ > 0.98). The standards were analyzed according to a previous adsorption step using the GFAD followed by thermal desorption and chromatographic separation procedures.

The identified compounds were classified in four groups. Thus, for instance, the analyzed bioethanol sample contained 56 ± 18 mg L^−1^ of hydrocarbons including from decane (C10) to nonadecane (C19). Alcohols, other than ethanol, were present at a concentration of 170 ± 51 mg L^−1^. As it has been previously reported, propan-2-ol uses to be present in bioethanol samples at hundreds of mg L^−1^ [36]. This fact was later verified by modifying the GC separation conditions, and a small peak of propan-2-ol was found on the tail of the wide ethanol signal. Another group of compounds found in the present study was ketones, not encountered when using GC-FID [36], likely because of the lower limit of detection obtained in the present work. The total concentration of these compounds was 40 ± 12 mg L^−1^. These data were also in agreement with previously published work based on the use of GC–MS without sample preconcentration [36]. Finally, substituted benzene aromatic compounds were encountered at a total content of 35 ± 10 mg L^−1^.

### Considerations on the suitability of using GAFDs for gaseous analytes adsorption–desorption

The findings presented within this paper hold profound significance for the realm of chemical analysis involving novel materials, thereby engendering intricate kinetic-technological implications. In the contemporary landscape, mere adherence to analytical benchmarks no longer suffices; instead, such analyses must harmonize with the imperatives of sustainable development. In this context, the pursuit of innovative, sustainable devices and techniques, often leveraging emergent functional materials, has become a topic of heightened interest.

The featured GFAD embodies a biphasic composition wherein the metal matrix phase provides amplified thermal conductivity, while the guest phases (comprising activated carbon particles) prove instrumental in adsorption/desorption processes of volatile organic compounds. This binary architecture imparts the GFAD with advantages that surpass those of its commercial counterpart, the Twister, renowned for its low thermal conductivity and restricted surface area. The implications of these differences are particularly noteworthy. As delineated in the “Results and discussion” section, the experimental conditions led to the GFAD and Twister manifesting temperature differences of under 1% concerning the programmed temperature along the container tube walls during the heating ramp. This outcome was predominantly attributed to factors such as low gas flow, a moderate heating ramp, and the extensive length of the gas line tube relative to the size of the sample. The elongated tube length facilitated the carrier gas reached the sample at the maximum programmed temperature (350 °C). Since the heating area (the entire length of the container tube) is too large, these conditions, which are favorable for proper material comparison, are not suitable for state-of-the-art energy efficiency requirements.

This section, therefore, retained the gas flow and heating ramp parameters while deploying a shorter tube (4 cm length) solely heated where the sample (1 cm length) resides. A constant heat flux of 2500 Wm^−2^ facilitated heating, with the intent of assessing the temporal temperature profile across the front and rear regions of the sorbent surfaces for both materials. Within these energy-restricted conditions, the temperature profiles of the two materials differ significantly (Fig. S4). Contrary to the Twister, which reaches 350 °C in the rear region in less than 200 s, the GFAD necessitates approximately 250 s to attain the same temperature. This discrepancy primarily arises from distinct modes of heat transfer between the walls of the heated container tube and the materials. While the functional surface of the Twister® is in direct physical contact with the heated container tube, the guest phases of the GFAD (constituting sorptive elements) gain heat through contact with the matrix phase, which, in turn, transfers heat via contact with the heated container tube walls. Despite the slower heating kinetics of GFAD, the temperature gradient between the front and rear zones remains significantly lower (6%) than in the Twister® (127%). This pronounced gradient disparity in the Twister® hinders the study of the desorption-temperature relationship due to the broad spectrum of desorption temperatures encompassed by the species within a given time frame. Visual insight into these differences is discernible in Fig. S4B and S4C, illustrating the temperature profiles of the studied configurations. Thus, it becomes patently evident that the utilization of GFADs boasts superior energy efficiency when contrasted with conventional Twisters, since they can achieve higher temperatures with diminished energy consumption. Furthermore, GFADs have substantial analytical potential, attributed to the combination of their properties, enabling the generation of minute thermal gradients conductive to the identification of species undergoing desorption within a narrow temperature range.

Throughout the present work, it was observed that the GFAD tubes could be reused without modification in their trapping and desorption capabilities after performing, at least, 100 analyses. This observation revealed the excellent stability of the novel developed sorbent for the determination of gaseous compounds.

## Conclusions

The applicability of a novel sorbent structure to perform studies of adsorption-thermal desorption of vapor compounds is demonstrated. The so-called Guefoam Adsorption Device (GFAD) has two main innovations against the existing devices: first, it contains a metallic net in which the adsorbing phase (*i.e.*, activated carbon) is dispersed; and second, the entire surface of carbon particles is exposed to the sample. As a consequence of both features, the GFAD leads to the following: (i) higher total available surface area than that for conventional devices that may improve the trapping efficiency, mainly for compounds present at high concentrations; (ii) higher robustness and thermal stability as compared to a PDMS coated bar; and (iii) an improved desorption efficiency directly linked to a higher thermal conductivity of the metallic foam than that for the Twister®. Computational simulations unequivocally showcase that the properties of GFADs facilitate chemical analysis with markedly diminished energy consumption, aligning with environmentally friendly conscientious and sustainable analytical methodologies.

The developed bars behave satisfactorily against a conventional Twister® in terms of sensitivity and limits of detection, especially for highly volatile compounds. In the case of less volatile species, similar results can be found with both approaches. Therefore, the new GFAD may be useful for determining light as well as heavy COVs through headspace sorptive extraction-thermal desorption gas chromatography mass spectrometry (HSSE-TD-GC–MS).

Additional studies should be carried out in order to determine the analytical figures of merit of the method as well as its accuracy for the determination of volatile compounds of interest for food and environmental analysis. This work is currently being performed in our laboratories and will be the subject of further reports.

### Supplementary Information

Below is the link to the electronic supplementary material.

Supplementary file1 (DOCX 1240 KB)

## Data Availability

Additional data are available as electronic supplementary materials.

## References

[1] Li Y, Wei M, Liu L, Yu B, Dong Z, Xue Q (2021). Evaluation of the effectiveness of VOC-contaminated soil preparation based on AHP-CRITIC-TOPSIS model. Chemosphere.

[2] Hrobonova K, Jablonsky H, Kralik M, Vizarova K (2023). Advanced sampling, sample preparation and combination of methods applicable in analysis of compounds in aged and deacidified papers. A minireview. J Cult Herit.

[3] Cecchi L, Migliorini M, Mulinacci N (2021). Virgin olive oil volatile compounds: Composition, sensory characteristics, analytical approaches, quality control, and authentication. J Agric Food Chem.

[4] Demeestere K, Dewull J, De Witte B, Van Langenhove H (2007). Sample preparation for the analysis of volatile organic compounds in air and water matrices. J Chromatogr A.

[5] Sánchez-Rojas F, Bosch-Ojeda C, Cano-Pavón JM (2009). A review of stir bar sorptive extraction. Chromatographia.

[6] Blomberg S, Roeraade J (1990). Improved thick film open tubular traps for the enrichment of volatile organic compounds from air and water. J High Resolut Chromatogr.

[7] David F, Ochiai N, Sandra P (2019). Two decades of stir bar sorptive extraction: A retrospective and outlook. Trends Anal Chem.

[8] Nongonierma A, Voilley A, Cayot P, Le Quéré JL, Springett M (2006). Mechanisms of extraction of aroma compounds from foods, using adsorbents. Effect of Various Parameters. Food Rev Int.

[9] Nogueira JMF (2015). Stir-bar sorptive extraction: 15 years making sample preparation more environment-friendly. Trends Anal Chem.

[10] Martin A, Margoum C, Coquery M, Random J (2016). Combination of sorption properties of polydimethylsiloxane and solid-phase extraction sorbents in a single composite material for the passive sampling of polar and apolar pesticides in water. J Sep Sci.

[11] Brown RH, Charlton J, Saunders KJ (1981). The development of an improved diffusive sampler. Am Ind Hyg Assoc J.

[12] Gallego E, Roca FJ, Perales JF, Guardino X (2011). Comparative study of the adsorption performance of an active multi-sorbent bed tube (Carbotrap, Carbopack X, Carboxen 569) and a Radiello® diffusive sampler for the analysis of VOCs. Talanta.

[13] Woolfenden E (2010). Sorbent-based sampling methods for volatile and semi-volatile organic compounds in air. Part 1: Sorbent-based air monitoring options. J Cromatogr A.

[14] UK Health and Safety Laboratory Methods for the determination of hazardous substances (MDHS) 53/2. 1,3-Butadiene in air (2003) Laboratory method using pumped samplers, thermal desorption and gas chromatography, 1–12. https://citeseerx.ist.psu.edu/

[15] Watson N, Bates M (2009) Evaluation of nanoparticles as a potential sorbent for thermal desorption. Markes International Internal Report (available from enquiries@markes.com )

[16] Bunte G, Hürttlen J, Pontius H, Hartlieb K, Krause H (2007). Gas phase detection of explosives such as 2,4,6-trinitrotoluene by molecularly imprinted polymers. Anal Chim Acta.

[17] Kabir A, Locatelli M, Ulusoy HI (2017). Recent trends in microextraction techniques employed in analytical and bioanalytical sample preparation. Separations.

[18] Kuchek HA (1964) Method for making foamlike mass of metal. United States Patent 3236706

[19] Carlson NG (1967) Cast porous metal. United States Patent 3210166

[20] Molina-Jorda JM (2017) oam materials with pores interconnected with guest phases, process for preparing these materials and uses thereof. Spanish Patent P201730890

[21] Molina-Jorda JM (2018) Foam materials with pores interconnected with guest phases, process for preparing these materials and uses thereof. PCT Patent PCT/ES2018/070474

[22] Molina-Jordá JM (2020). Highly adsorptive and magneto-inductive Guefoams (multifunctional guest-containing foams) for enhanced energy- efficient preconcentration and management of VOCs. Appl Mater Interfaces.

[23] Maiorano LP, Molina JM (2018). Challenging thermal management by incorporation of graphite into aluminium foams. Mater Des.

[24] Yin X, Huang J, Huang J, Wu W, Tong T, Liu S, Zhou L, Liu Z, Zhang S (2022). Identification of volatile and odor-active compounds in Hunan black tea by SPME/GC-MS and multivariate analysis. LWT.

[25] Fang Z, Li C, Gu Y, Wen C, Ye H, Ma J, Liang Z, Yang L, Wu J, Chen H (2022). Flavour analysis of different varieties of camellia seed oil and the effect of the refining process on flavour substances. LWT.

[26] Nielsen AT, Jonsson S (2002). Trace determination of volatile sulfur compounds by solid-phase microextraction and GC–MS. Analyst.

[27] Balasubramanian S, Panigrahi S (2011). Solid-phase microextraction (SPME) techniques for quality characterization of food products: A review. Food Bioprocess Technol.

[28] Saito Y, Ueta I, Kotera K, Ogawa M, Wada H, Jinno K (2006). In-needle extraction device designed for gas chromatographic analysis of volatile organic compounds. Talanta.

[29] Zhou X, Yan Z, Zhou X, Wang C, Liu H, Zhou H (2022). An assessment of volatile organic compounds pollutant emissions from wood materials: A review. Chemosphere.

[30] Barba C, Thomas-Danguin T, Guichard E (2017). Comparison of stir bar sorptive extraction in the liquid and vapour phases, solvent-assisted flavour evaporation and headspace solid-phase microextraction for the (non)-targeted analysis of volatiles in fruit juice. LWT.

[31] Paulsen PD, Cannon FS (1999). Polytherm model for methylisobutylketone adsorption onto coconut-based granular activated carbon. Carbon.

[32] Prado C, Alcaraz MJ, Fuentes A, Garrido J, Periago JF (2006). Storage stability of ketones on carbon adsorbents. J Chromatogr A.

[33] Caven-Quantrill DJ, Buglass AJ (2006). Comparison of micro-scale simultaneous distillation–extraction and stir bar sorptive extraction for the determination of volatile organic constituents of grape juice. J Chromatogr A.

[34] Liu PKT, Feltch SM, Wagner NJ (1987). Thermal desorption behavior of aliphatic and aromatic hydrocarbons loaded on activated carbon. Ind Eng Chem Res.

[35] Sánchez C, Santos S, Sánchez R, Lienemann CP, Todolí JL (2020). Profiling of organic compounds in bioethanol samples of different nature and the related fractions. ACS Omega.

[36] Habe H, Shinbo T, Yamamoto T, Sato S, Shimada H, Sakaki K (2013). Chemical analysis of impurities in diverse bioethanol samples. J Jpn Petrol Inst.

