# Supplementary material for: Evaluation of highly adsorptive Guefoams (multifunctional guest-containing foams) as a potential sorbent for determination of volatile organic compounds (VOCs) by means of thermal desorption
Source: Mikrochim Acta. 2024 Feb 29;191(3):169. doi: 10.1007/s00604-024-06249-9 (PMC10904424; doi:10.1007/s00604-024-06249-9)
Supplement: Supplementary file 1 — Supplementary file1 (DOCX 1240 KB) [file 604_2024_6249_MOESM1_ESM.docx]

**Supplementary Information**

**Evaluation of highly adsorptive Guefoams (multifunctional guest-containing foams) as a potential sorbent for analysis of volatile organic compounds (VOCs) by means of thermal desorption**

**Raquel Sánchez^1#^, Ana Beltrán Sanahuja^1#^, Lucila Paola Maiorano Lauría^2^,**

**José Luis Todolí^1^*, José Miguel Molina Jordá^2^**

^1^Analytical Chemistry, Nutrition and Food Sciences Department, University of Alicante, P.O. Box 99, 03080, Alicante, Spain.

^2^Inorganic Chemistry Department, University of Alicante, P.O. Box 99, 03080, Alicante, Spain.

Corresponding email: jose.todoli@ua.es

|  | 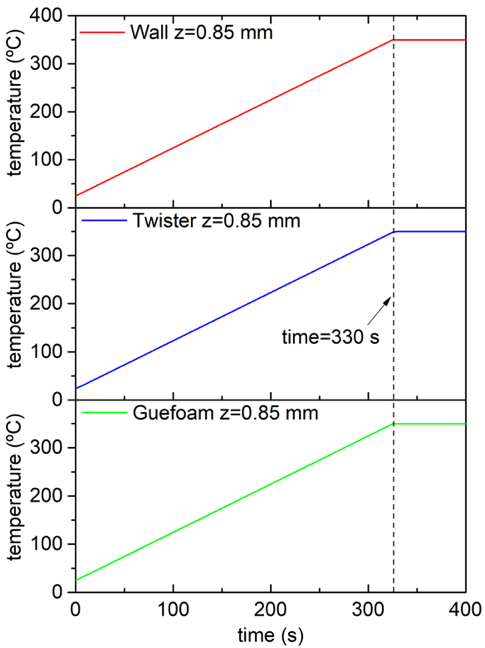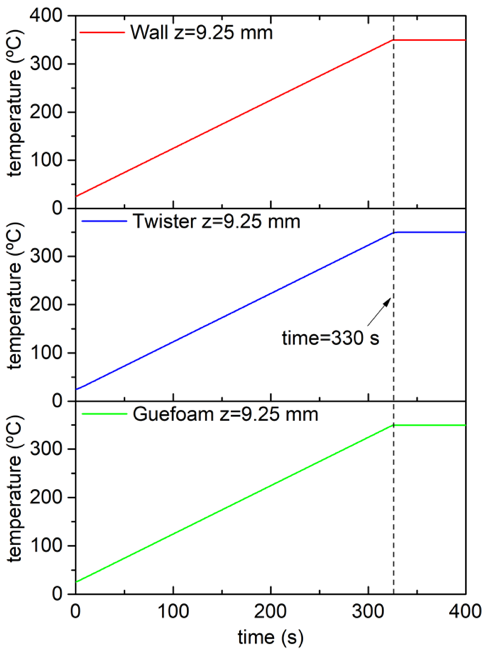 |  |
| --- | --- | --- |

*Fig. S1. Time evolution of the temperature in the front and rear regions of each material under the experimental operating conditions during the temperature rise. The programmed temperature of the container tube is shown for comparison.*

|  | 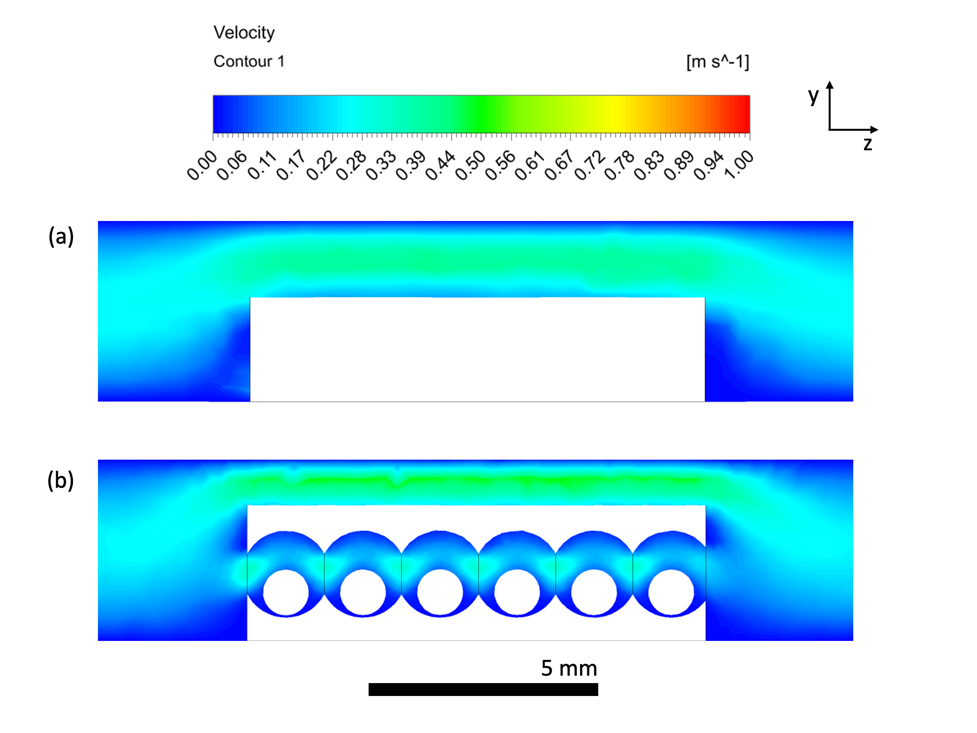 |  |
| --- | --- | --- |

*Fig. S2. Velocity profiles of the fluid (helium) in the container tube in the vicinity of the materials after reaching the maximum temperature (350 ºC).*


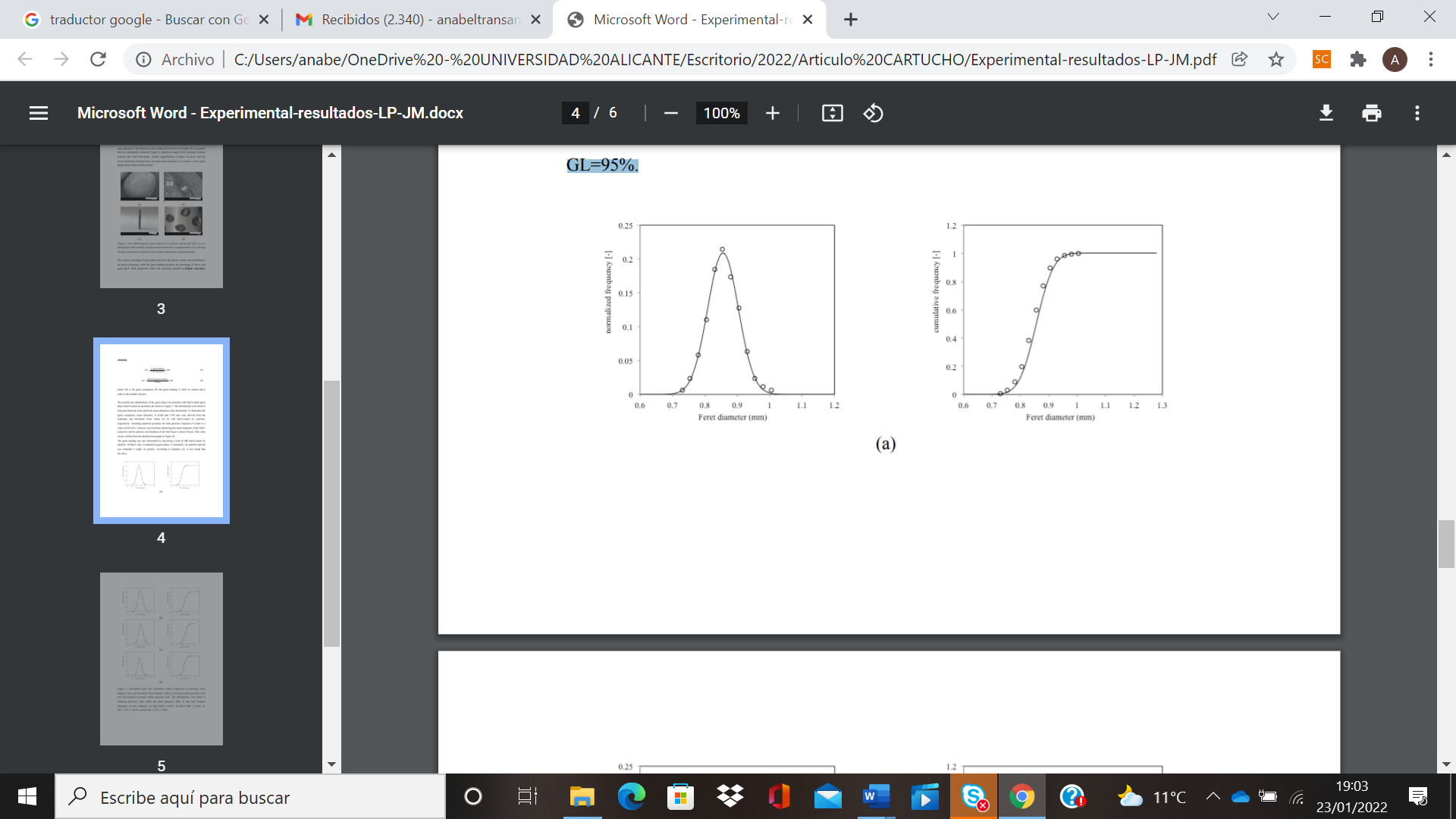


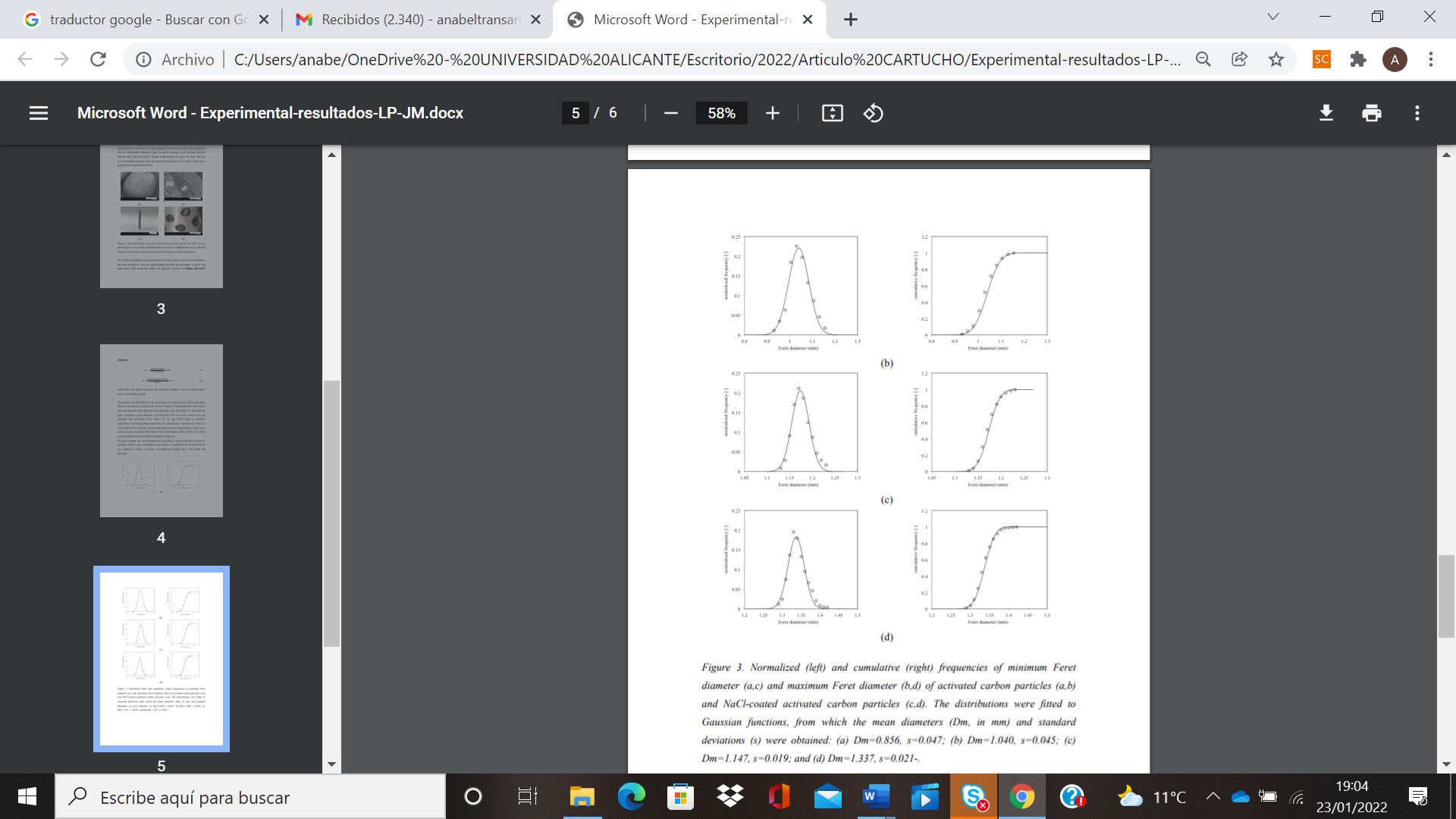


*Fig. S3. Normalized (left) and cumulative (right) frequencies of minimum Feret diameter (A,C) and maximum Feret diameter (B,D) of activated carbon particles (A,B) and NaCl-coated activated carbon particles (C,D). The distributions were fitted to Gaussian functions, from which the mean diameters (Dm, in mm) and standard deviations (s) were obtained: (a) Dm=0.856, s=0.047; (b) Dm=1.040, s=0.045; (c) Dm=1.147, s=0.019; and (d) Dm=1.337, s=0.021.*


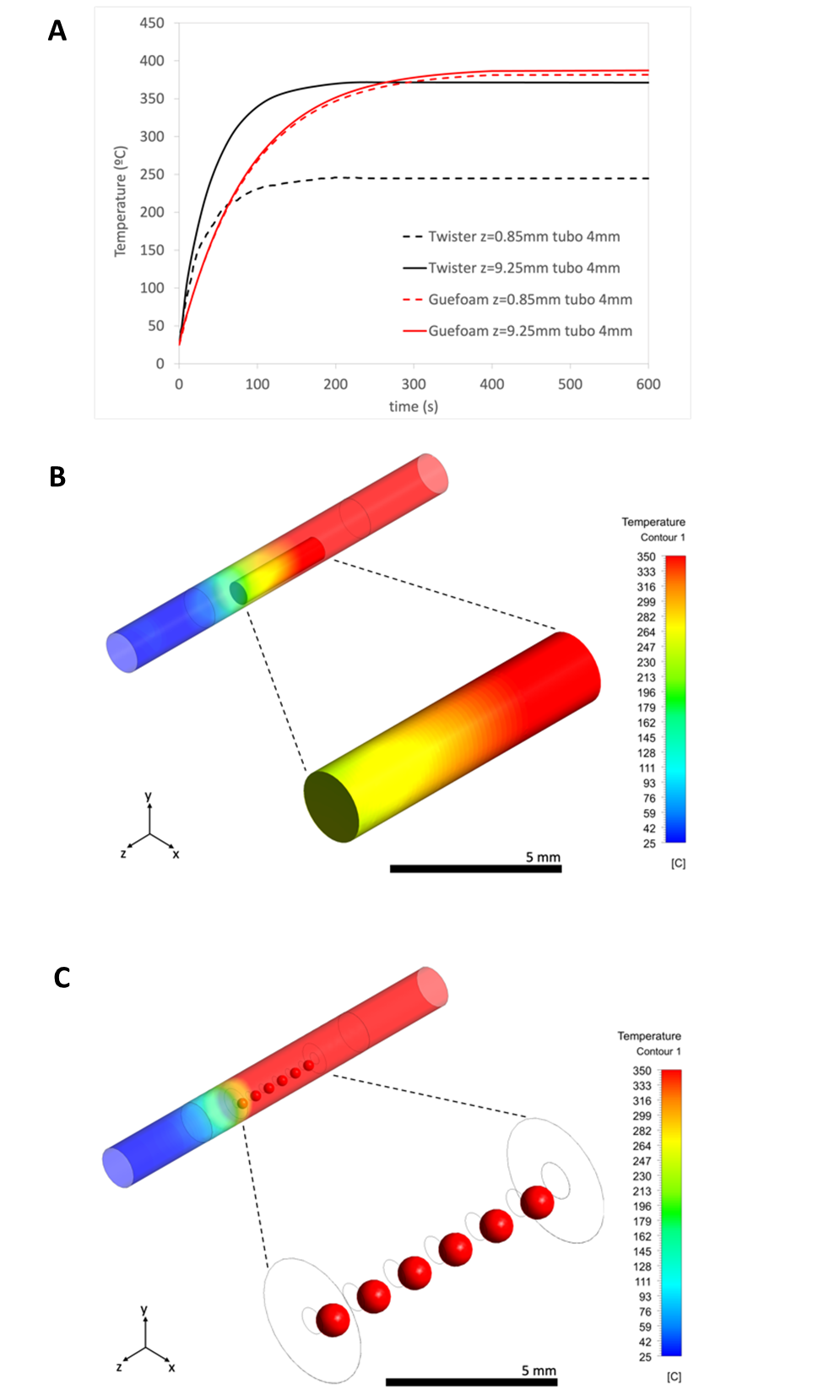


*Fig. S4. (A) Temperature over time profiles of the front (z = 0.85 mm) and back (z = 9.25 mm) regions of Twister and GFAD for 2500 Wm^-2^ of heat flux; temperature profiles of the container tube and detailed temperature profiles of Twister (B) and guest phases in Guefoam (C) under steady-state conditions (t > 500 s).*

*Table S1. Organic compounds present in the studied solutions 1 and 2 and their respective boiling points (ºC) and retention time (min).*

| **Solution 1: Organic compound** | **Boiling point (ºC)** | **Retention time (min)** |
| --- | --- | --- |
| Ethanol | 78 | 2.32 |
| Propan-2-ol | 97 | 5.10 |
| Toluene | 111 | 9.97, 10.22, 10.38, 13.35 |
| Xylene | 138-144 | 18.48 |
| **Solution 2: Organic compound** | **Boiling point (ºC)** | **Retention time (min)** |
| Acetone | 56 | 2.81 |
| Dichloromethane | 39.6 | 3.29 |
| MIBK | 116 | 5.49 |
| Decane | 174 | 24.64 |
| Dodecane | 216 | 29.74 |
| Tetradecane | 254 | 31.47 |

*Table S2. List of organic compounds found in the bioethanol analyzed sample.*

| **Retention time (min)** | **Relative peak area** | **Compound** |
| --- | --- | --- |
| ***Most abundant compounds*** | | |
| 2.56 | 24.49 | Ethanol |
| 19.71 | 2.88 | 3,6-Dimethyldecane |
| 20.80 | 1.28 | + 3,6-Dimethyldecane  + Nonane, 5-methyl-5-propyl- |
| 24.15 | 6.67 | 1,3-bis(1,1-dimethylethylBenzene |
| 24.21 | 1.82 | 2,3,5-trimethyl-Decane |
| 28.65 | 1.13 | Substituted Phenol |
| 31.94 | 1.02 | (3-Thio-2-benzo[B]thienylidene)aniline |
| 33.04 | 1.04 | Benzoic acid |
| 33.47 | 1.13 | Hexadecanoic acid, ethyl esther |
| 33.71 | 1.12 | Cholesta-7,9(11)-dien-3-ol |
| 38.39 | 1.05 | Nonadecane |
| 41.16 | 1.26 | Tetracosane |
| ***Trace compounds*** | | |
| 20.25 | 0.41 | 2-ethyl-1-Hexadecanol |
| 21.09 | 0.33 | Phenol |
| 22.98 | 0.22 | Undecane, 2,4-dimethyl- |
| 23.19 | 0.35 | Undecane, 4,8-dimethyl- |
| 23.46 | 0.13 | Undecane, 4,6-dimethyl- |
| 23.83 | 0.23 | Tridecane, 2,5-dimethyl- |
| 24.34 | 0.43 | Pentadecane |
| 24.43 | 0.37 | Tetradecane |
| 24.49 | 0.34 | Tridecane |
| 24.64 | 0.19 | Decane |
| 25.33 | 0.31 | Benzaldehyde, 4-propyl- |
| 26.88 | 0.23 | Octadecane |
| 27.05 | 0.38 | 1,1'-Biphenyl, 2,2'-dimethyl- |
| 27.66 | 0.17 | 1,1'-Biphenyl, 2-ethyl- |
| 27.95 | 0.23 | Tricosane |
| 28.56 | 0.19 | 2-Propanone, 1,1-diphenyl- |
| 30.03 | 0.14 | Nonadecane |
| 31.72 | 0.24 | 1-Hexadecanol |
| 31.88 | 0.28 | Dodecanenitrile |
| 35.51 | 0.37 | Cholesta-7,9(11)-dien-3-ol |
| 36.23 | 0.33 | Tetradecane, 2,6,10-trimethyl- |
